# Genetic testing of 248 Chinese aortopathy patients using a panel assay

**DOI:** 10.1038/srep33002

**Published:** 2016-09-09

**Authors:** Hang Yang, Mingyao Luo, Yuanyuan Fu, Yandong Cao, Kunlun Yin, Wenke Li, Chunjie Meng, Yanyun Ma, Jing Zhang, Yuxin Fan, Chang Shu, Qian Chang, Zhou Zhou

**Affiliations:** 1 State Key Laboratory of Cardiovascular Disease, Beijing Key Laboratory for Molecular Diagnostics of Cardiovascular Diseases, Diagnostic Laboratory Service, Fuwai Hospital, National Center for Cardiovascular Diseases, Chinese Academy of Medical Sciences and Peking Union Medical College, Beijing, 100037, China; 2 State Key Laboratory of Cardiovascular Disease, Center of Vascular Surgery, Fuwai Hospital, National Center for Cardiovascular Diseases, Chinese Academy of Medical Sciences and Peking Union Medical College, Beijing, 100037, China; 3 Analyses Technologies, Beijing, 100102, China; 4 John Welsh Cardiovascular Diagnostic Laboratory, Department of Pediatrics, Baylor College of Medicine, Houston, TX, 77030, USA

## Abstract

Inherited aortopathy, which is characterized by a high risk of fatal aortic aneurysms/dissections, can occur secondarily to several syndromes. To identify genetic mutations and help make a precise diagnosis, we designed a gene panel containing 15 genes responsible for inherited aortopathy and tested 248 probands with aortic disease or Marfan syndrome. The results showed that 92 individuals (37.1%) tested positive for a (likely) pathogenic mutation, most of which were *FBN1* mutations. We found that patients with a *FBN1* truncating or splicing mutation were more prone to developing severe aortic disease or valvular disease. To date, this is the largest reported cohort of Chinese patients with aortic disease who have undergone genetic testing. Therefore, it can serve as a considerable dataset of next generation sequencing data analysis of Chinese population with inherited aortopathy. Additionally, according to the accumulated data, we optimized the analysis pipeline by adding quality control steps and lowering the false positive rate.

Inherited aortopathy, which is characterized by aortic dilation or aortic aneurysms/dissection, may be syndromic, as occurs in Marfan syndrome (MFS)^1^, Loeys-Dietz syndrome (LDS)^2^, Ehlers-Danlos syndrome, vascular type (vEDS)^3^, and Shprintzen-Goldberg syndrome (SGS)^4^, or non-syndromic, in which abnormalities are restricted to the aorta^5^. Although these diseases have their own unique characteristics, they also share some clinical manifestations, which makes the precise diagnosis and treatment strategy difficult. Previous studies demonstrated that the mortality after the rupture of thoracic aortic aneurysms (TAA) was as high as 97%, with a median survival time of 3 days^6^, and the acute aortic dissection patients had a higher re-intervention rate, even if they survived the initial surgery^7^. Hence, early diagnosis is important because it provides valuable time for prophylactic measures to be taken. Genetic testing can help to detect the pathogenic genes/mutations involved in the disease and confirm the diagnosis before the full development of symptom, thereby reduce the rate of cardiovascular events.

Several causative genes for syndromic aortopathy have been identified, including *FBN1* for Marfan syndrome^8^, *TGFBR1/2, SMAD3, TGFB2* for Loeys-Dietz syndrome^9^,^10^,^11^, *COL3A1* for Ehlers-Danlos syndrome, vascular type^12^, and *SLC2A10* for arterial tortuosity syndrome^13^. Additionally, an increasing number of genes have been implicated in the pathogenesis of thoracic aortic aneurysms, including *MYH11, ACTA2, NOTCH1, MYLK, PRKG1,* and *SKI*^14^.

The clinical utility of genetic testing for heritable aortopathy is now well established^15^,^16^, and several commercial panel tests containing different numbers of genes are available. However, due to the lack of a database for Chinese population, it is challenging to determine the pathogenicity of genetic variants for Chinese patients. To identify genetic mutations and make a precise diagnosis and to establish an aortopathy genetic database for Chinese population, we recruited 248 probands with aortic disease or Marfan syndrome in Fuwai hospital and performed gene panel testing involving 15 genes related to inherited aortopathy. Herein, we report the molecular findings from the 248 patients, which, at present, is the largest group of aortic disease patients ever reported in China. Further, we optimized the analysis pipeline by adding quality control steps and lowering the false positive rate.

## Results

### Aortopathy panel performance

Sequencing of the 15 aortopathy genes (Table 1) in the 248 samples yielded a mean depth of ~350X and coverage of 98.7% (Supp. Figure S1). Exons in *FBN1* with low (<20X) or no coverage were subject to Sanger sequencing to obtain 100% coverage. In addition, potential pathogenic mutations and rare variant of unknown significance (VUS) were confirmed using Sanger sequencing.

### Automated and optimal analysis pipeline

Initially, we used Ion Torrent Suite and Ion Reporter, which were provided by Life Tech, to accomplish the alignment, variant calling and annotation processes. Based on our growing data and experience, we developed an automated and optimal analysis plugin named iAorta, which allowed us to automatically pick up suspected pathogenic mutations or VUS from polymorphism or false-positive variants, add quality control steps to assess the sequencing quality and indicate possible false-negative variants, and remove frequent false-positive mutations based on our 248 samples. Compared with Ion Torrent Suite, the analysis strategy of iAorta was to relax the filter conditions to avoid false negative variants. Subsequently, according to the accumulated data, false positive variants were removed.

After re-analyzing the sequencing data from our 248 samples by iAorta, we obtained a false-negative list (Supp. Table S1). We also generated a false-positive list after validation by Sanger sequencing. Most of the false-positive mutation were scattered over the end of amplicon and were likely introduced by degenerate primers and mapping error. For frameshift/non-frameshift indels, we evaluated the confidence based on the coverage depth (>20x), allele frequency (>10%), and strand bias (both forward and reverse allele reads >3, both forward/reverse and reverse/forward >0.7). If any of the three conditions was not satisfied, the sample was classified as a possible false-positive mutation and marked as “DropIndel”. After we removed some frequent false-positive mutations (Supp. Table S2) and modified our analysis pipeline, the false-positive rate decreased from 25.4% to 15.4%.

In addition, all of the bases coding cysteine in *FBN1* were assigned as a “hotspot”. When there was a “NoCall” in the position, an alert for a possible false negative region was generated, and the exon was then Sanger sequenced.

### Molecular findings of the aortopathy cohort

A total of 248 patients (162 males and 86 females) with Marfan syndrome and its related aortic diseases, were enrolled in our cohort, with a mean age of 46 years (5–60 years). The primary clinical diagnoses of these probands submitted for aortopathy panel testing were summarized in Table 2. Among the 248 individuals, 92 (37.1%) were tested positive for a (likely) pathogenic mutation, 70 (28.2%) had a VUS, and 86 (34.7%) were tested negative using the 15-gene aortopathy panel. Most of the (likely) pathogenic mutations were located in the *FBN1* gene, because the cysteine residues in this gene were evolutionarily conserved and had essential functions^17^. Accordingly, the destruction or generation of a cysteine residue suggested that the mutation was probably pathogenic^18^. The pathogenicity of missense mutations in other genes was difficult to define due to the lack of functional studies or strong family segregation evidence. (Likely) pathogenic mutations were identified in *FBN1*, *TGFBR1/2, ACTA2, MYH11, COL3A1* and *SLC2A10* (Table 3), and VUS were identified in all 15 genes in the panel.

A genotype-phenotype correlation between *FBN1* mutation type and aortic events was also investigated. Of all the 248 probands, 82 were tested positive for a (likely) pathogenic *FBN1* mutation. Among them, 28 had undergone surgery due to a life-threatening aortic dissection, 21 had undergone prophylactic surgery due to aortic aneurysm, 6 had a valve replacement due to severe valvular disease, 4 had mild aortic dilation and came for genetic testing because of other system manifestations in Marfan syndrome, and the remaining 24 patients had no complete clinical information. We attempted to study the correlation between *FBN1* mutation type and severity of aortic events, and the results were listed in Table 4. Among patients with a *FBN1* truncating or splicing mutation, 15 suffered from life-threatening aortic dissection, 5 had severe valvular disease, while 9 had aortic aneurysm and therefore underwent prophylactic surgery. Besides, 3 patients with one *FBN1* truncating or splicing mutation only showed mild aortic dilation probably due to a young age, therefore they were not excluded to have aortic disease progression in the future. Additionally, in the aneurysm group, patients with a *FBN1* truncating or splicing mutation took a prophylactic surgery at a younger age (25.6y vs. 33.4y) than those with a missense mutation. These results suggested that patients with *FBN1* truncating or splicing mutation were more prone to developing severe aortic disease or valvular disease.

### Variant reclassification

When available, family segregation studies were performed to assist in the variant classification. In this study, 18 variants were reclassified through the family segregation study in our patient cohort (Table 5).

The *FBN1*, c.1427G>A (p.Cys476Tyr) variant in case AD246, which presented a classic MFS phenotype and a positive family history, was originally classified as likely pathogenic. However, it was downgraded to VUS after familial targeted sequencing revealed that the variant was not present in his affected sibling. Thus, whole exome sequencing (WES) was performed in the proband and his two affected sisters to find other potential pathogenic mutations. Another interesting case was as reported in our previously published paper^19^. The variant *TGFBR2*, c.1142G>C (p.Arg381Pro) was detected in a 5 year-old boy, who had a distinctive LDS phenotype of descending pseudoaneurysm, artery tortuosity, bifid uvula, hypertelorism. However, the mutation was also carried by his healthy father, which made its pathogenicity doubtful, although it was once reported as pathogenic in a LDS patient^20^. Further functional study was necessary to confirm its pathogenicity.

## Discussion

Genetic testing is important for the early and accurate diagnosis of diseases. Although patients with Marfan syndrome and its related diseases are all characterized with aortopathy, they may differ in their progress of aortic aneurysm/dissection. It was previously reported that LDS patients had more aggressive arterial disease and rupture, with a median survival time of only 26 years^2^, compared with 48 years for patients with vEDS^3^ and 70 years for those with MFS^21^. However, with early diagnosis and proper management, LDS was particularly amendable to treatment. The incidence of fatal intraoperative or postoperative complications with vascular surgery was only 1.7% in LDS^2^ compared with approximately 45% in vEDS^3^.

The NGS (next generation sequencing)-based assays for screening inherited aortopathy genes have been well established and utilized in some laboratories^15^,^16^. Sequencing data processing and analysis is the key point, and validating the candidate causal variants via Sanger sequencing is the most time-consuming step. Therefore, how to optimize the algorithms to lower the false-positive rate without raising the false-negative rate is extremely important. In our study, we developed an automated and optimized pipeline named iAorta that automatically accomplished read mapping, recalibration, quality control, alignment, variant calling, annotation and variant filtering. Compared to the Ion Torrent Suite and Ion Reporter software, which were provided by Life Tech, iAorta was used more flexibly, which allowed us to automatically pick up suspected pathogenic mutations and VUS from polymorphism or false-positive variants, add quality control steps to assess the sequencing quality and to indicate possible false-negative variants, remove frequent false-positive mutations based our existing data and drop the low confidence indel variants to reduce the false-positive rate.

In addition to data processing and analysis, the classification of variant pathogenicity is challenging. Novel variants should be subjected to functional studies, but these are costly, time consuming, and often impractical in the clinical setting. Therefore, classification is largely dependent on database knowledge, which is extremely deficient in Chinese populations. The aim of our study was to build the largest shared database for Chinese aortopathy patients. In our cohort, 92 patients (37.1%) tested positive for a (likely) pathogenic mutation, including 84 Marfan patients, as well as 3 LDS, 3 TAAD (thoracic aortic aneurysms and aortic dissection), 1 vEDS and 1 arterial tortuosity syndrome case. Additionally, the results of the patients’ family members were helpful for pathogenicity classification. Specifically, in our study, 18 variants were reclassified based on family segregation studies. After screening by the current gene panel testing, some cases remained negative, although they presented classical clinical phenotypes or family histories. As a follow-up, we intend to perform MLPA (Multiplex Ligation-dependent Probe Amplification) or WES on these samples to find large deletion/duplication or new potential causative genes. Besides, in more than one third of the patients, no suspected mutation was identified, which suggested that additional aortopathy genes might exist. We anticipate that clinical sensitivity will rise as additional genes are identified and included in the panel and that VUS can be reclassified with increasing numbers of samples and family segregation studies. Several recently identified TAA genes, such as *TGFB3*^22^, *MFAP5*^23^, *MAT2A*^24^ and *LOX*^25^, can be added to the gene list.

A genotype-phenotype correlation between *FBN1* mutation type and aortic events was investigated. Interestingly, we found that patients with a *FBN1* truncating or splicing mutation were more prone to developing severe aortic disease or valvular disease than the patients with a *FBN1* missense mutation. Similarly, Baudhuin *et al*. once reported that a higher frequency of truncating or splicing *FBN1* variants was observed in MFS patients with an aortic event than in those without a reported aortic event^26^. However, the mechanism whereby *FBN1* truncating or splicing mutations exert their effect on aneurysm progression and severity is not clear, which deserves our further investigation.

In summary, our data further expands the *FBN1* mutation spectrum and offer evidence for the genotype-phenotype correlation given that Marfan patients with a *FBN1* truncating or splicing mutation are more prone to developing severe aortic disease or valvular disease. The aortopathy panel assay undoubtedly presents a highly valuable clinical tool and lays the foundation for further study. We are dedicated to constructing the largest Chinese aortopathy genetic database and continually improving our testing quality.

## Materials and Methods

### Patients and consent

The study was approved by the ethics committee of Fuwai hospital and adhered to the Declaration of Helsinki. All experimental protocols were approved by the ethics committee of Fuwai hospital, and were carried out in accordance with the approved guidelines. All of the patients enrolled in this study were referred by the center of vascular surgery in Fuwai hospital. Each individual accepting the genetic test was adequately informed regarding the benefits and risks of the test and signed the consent form.

Between Feb 2014 and Apr 2016, we tested a total of 248 patients with various aortic phenotypes, such as early onset aortopathy patients with no apparent secondary causes and (suspected) Marfan patients. The follow-up study was carried out in subsequent clinic visits to the outpatient department and by telephone interviews.

### Gene panel testing

A custom-designed gene panel containing 15 genes known to be associated with Marfan syndrome and its related aortic diseases was ordered from Life Tech, USA. The size of the panel was 168.67 kb, with coverage of 99.39% of the target regions.

Genomic DNA (deoxyribonucleic acid) was extracted from EDTA (eathylene diamine tetraacetic acid)–anticoagulated whole blood, and checked to assure the quality and quantity before processing. Library preparation was performed according to the manufacturer’s instructions (Ion AmpliSeq^TM^ library kit 2.0, Life Technologies, Inc.). Pooled libraries (up to 12–15 samples per chip) were sequenced on the Ion 318^TM^ Chip on Life PGM^TM^ instrument.

Suspected pathogenic variants and VUS were confirmed using Sanger sequencing. Exons in *FBN1* with low (<20X) or no coverage were also subjected to Sanger sequencing to obtain 100% coverage.

### Bioinformatics analysis

To perform the analysis automatically, the iAorta plugin was developed based on the sequencing platform for ion torrent PGM™. The pipeline consisted of read mapping, recalibration, quality control, variant calling, annotation and variant filtering. The annotation included genetic reference sequences, genomic and cDNA positions, amino acid changes, and related information available from public databases, such as 1000 Genomes, dbSNP142 (National Center for Biotechnology Information, http://www.ncbi.nlm.nih.gov/SNP/), NHLBI Grand Opportunity Exome Sequencing Project (ESP6500) (https://esp.gs.washington.edu/drupal/), ExAC03 (http://exac.broadinstitute.org), ClinVar, DrugBank, Online Mendelian Inheritance in Man (OMIM), Uniprot (http://www.uniprot.org), and the Human Gene Mutation Database (HGMD), Pfam (http://pfam.xfam.org). The *in-silico* based computation analysis was carried out using a suite of bioinformatics tools, including SIFT, Polyphen2, MutationTaster, Grantham, and PolyP.

Because most of the heritable aortopathies were autosomal-inherited rare Mendelian disease, the variants with a minor allele frequency (MAF) > 1% in the following databases were filtered out: the 1000 Genomes, ESP6500, ExAC03.

### Variant classification

Variants were analyzed for pathogenicity according to the recommendations from the American College of Medical Genetics (ACMG). Specifically, the analysis was based on the following criteria: (i) whether they were previously reported by functional study or family segregation study; (ii) the nature of the variant (e.g., nonsense, frameshift indel, or splicing mutations (intron ±1 or ±2)); (iii) variant frequency in the 1000 Genomes, Exome Sequencing Project (ESP6500) and ExAC03; (iv) conservation of the altered residue; (v) in-silico based computational prediction (SIFT, PholyPhen2, or MutationTaster); (vi) de novo mutation; and (vii) family segregation studies. Based on this information, a variant was classified into one of the 5 following categories: benign, likely benign, unknown significance, likely pathogenic or pathogenic^27^.

## Additional Information

**How to cite this article**: Yang, H. *et al*. Genetic testing of 248 Chinese aortopathy patients using a panel assay. *Sci. Rep.* **6**, 33002; doi: 10.1038/srep33002 (2016).

## Supplementary Material

Supplementary Information

## Figures and Tables

**Table 1:** Aortopathy panel genes.

Gene	Locus	Protein	Disease	Exons	Amplicons	Coverage
*ACTA2*	10q22–q24	actin, alpha 2, smooth muscle, aorta	TAAD	10	15	1
*COL3A1*	2q31	collagen, type III, alpha 1	vEDS	51	66	0.998
*FBN1*	15q21.1	fibrillin 1	Marfan, MASS, Mitral valve prolapse syndrome, Ectopia lentis syndrome, SGS	66	106	1
*FBN2*	5q23–q31	fibrillin-2	CCA	65	99	1
*MYH11*	16p13.13–p13.12	myosin-11	TAAD	43	61	0.971
*MYLK*	3q21	myosin light chain kinase, smooth muscle	TAAD	34	66	0.983
*NOTCH1*	9q34.3	neurogenic locus notch homolog protein 1	TAAD	34	78	0.892
*PRKG1*	10q11.2	cGMP-dependent protein kinase 1	TAAD	21	23	0.934
*SKI*	1p36.33	ski oncogene	SGS	8	19	0.971
*SLC2A10*	20q13.1	solute carrier family 2, facilitated glucose transporter member 10	Arterial tortuosity syndrome	8	26	0.977
*SMAD3*	15q22.33	mothers against decapentaplegic homolog 3	LDS, TAAD	13	46	0.922
*SMAD4*	18q21.1	mothers against decapentaplegic homolog 4	TAAD	12	54	1
*TGFB2*	1q41	transforming growth factor beta-2	LDS	8	36	0.922
*TGFBR1*	9q33–q34	TGF-beta receptor type-1	LDS,TAAD	11	39	1
*TGFBR2*	3p22	TGF-beta receptor type-2	LDS, TAAD	9	29	1

LDS, Loeys-Dietz syndrome; SGS, Shprintzen-Goldberg syndrome; TAAD, Thoracic aortic aneurysms and aortic dissection; MASS, The acronym MASS stands for mitral valve prolapse, myopia, borderline and non-progressive aortic enlargement, and nonspecific skin and skeletal findings that overlap with those seen in Marfan syndrome; vEDS, Ehlers-Danlos syndrome, vascular type; CCA, Congenital contractural arachnodactyly.

**Table 2:** Summary of primary diagnosis and genetic results of 248 probands in our cohort.

Primary Diagnosis	Cases	Genetic Results		
		(Likey) Pathogenic	VUS	No suspected variant
Marfan syndrome	65	55	5	5
Suspected Marfan syndrome	52	29	7	16
Suspected Loeys-Dietz syndrome	10	3	7	0
Non-syndromic aortic events	121	5	51	65
Total	248	92	70	86

**Table 3:** (Likely) Pathogenic mutations and VUS detected in our cohort.

Gene	Transcript	Exon/Intron	Nucleotide change	Protein change	De novo	Pathogenicity	Report Ref (PMID)
*ACTA2*	NM_001613	exon7	c.773G>A	p.Arg258His	NA	Likely Pathogenic	19409525
*ACTA2*	NM_001613	exon2	c.116G>A	p.Arg39His	NA	Likely Pathogenic	19409525
*COL3A1*	NM_000090	exon41	c.2932G>C	p.Gly978Arg	NA	Likely Pathogenic	
*FBN1*	NM_000138	exon33	c.4022A>G	p.Asn1341Ser	NA	Likely Pathogenic	10464652
*FBN1*	NM_000138	exon17	c.2055C>G	p.Cys685Trp	NA	Likely Pathogenic	12203987
*FBN1*	NM_000138	intron55	c.6740-1G>A		De novo	Pathogenic	
*FBN1*	NM_000138	exon47	c.5788G>C	p.Asp1930His	NA	Likely Pathogenic	17657824
*FBN1*	NM_000138	exon29	c.3496T>C	p.Cys1166Arg	NA	Likely Pathogenic	
*FBN1*	NM_000138	exon28	c.3440_3441insTTCAGCTGTC	p.Ser1147fs	NA	Pathogenic	
*FBN1*	NM_000138	exon40	c.4897_4898insCGCT	p.Cys1633fs	NA	Pathogenic	
*FBN1*	NM_000138	intron55	c.6739+1G>T		NA	Pathogenic	
*FBN1*	NM_000138	exon33	c.3995delA	p.Asn1332fs	Inherited from mother	Pathogenic	
*FBN1*	NM_000138	exon64	c.7871A>C	p.Asn2624Thr	NA	Likely Pathogenic	19293843
*FBN1*	NM_000138	intron13	c.1589-1G>A		NA	Pathogenic	
*FBN1*	NM_000138	exon54	c.6569G>A	p.Cys2190Tyr	NA	Likely Pathogenic	
*FBN1*	NM_000138	exon61	c.7477C>T	p.Gln2493Ter	NA	Pathogenic	
*FBN1*	NM_000138	exon7	c.643C>T	p.Arg215Ter	NA	Pathogenic	11139245
*FBN1*	NM_000138	exon58	c.7039_7040del	p.Met2347fs	NA	Pathogenic	
*FBN1*	NM_000138	exon37	c.4527dupT	p.Ile1510fs	NA	Pathogenic	
*FBN1*	NM_000138	exon13	c.1481G>A	p.Cys494Tyr	NA	Likely Pathogenic	24501682
*FBN1*	NM_000138	exon66	c.8525_8529del	p.Leu2842fs	Inherited from mother	Pathogenic	
*FBN1*	NM_000138	exon28	c.3352C>T	p.Gln1118Ter	De novo	Pathogenic	
*FBN1*	NM_000138	exon42	c.5162G>A	p.Cys1721Tyr	NA	Likely Pathogenic	9399842
*FBN1*	NM_000138	exon37	c.4532G>T	p.Cys1511Phe	De novo	Likely Pathogenic	
*FBN1*	NM_000138	exon40	c.4831delC	p.Gln1611fs	NA	Pathogenic	
*FBN1*	NM_000138	exon62	c.7606G>A	p.Gly2536Arg	NA	Likely Pathogenic	11524736
*FBN1*	NM_000138	exon44	c.5372G>A	p.Cys1791Tyr	NA	Likely Pathogenic	11700157
*FBN1*	NM_000138	exon63	c.7754T>C	p.Ile2585Thr	NA	Likely Pathogenic	10464652
*FBN1*	NM_000138	exon64	c.7955G>A	p.Cys2652Tyr	NA	Likely Pathogenic	17627385
*FBN1*	NM_000138	exon13	c.1585C>T	p.Arg529Ter	NA	Pathogenic	17663468
*FBN1*	NM_000138	exon31	c.3778G>T	p.Glu1260Ter	NA	Pathogenic	10464652
*FBN1*	NM_000138	exon58	c.7010_7011delinsCAC	p.Gly2337fs	NA	Pathogenic	
*FBN1*	NM_000138	exon50	c.6071G>A	p.Cys2024Tyr	NA	Likely Pathogenic	
*FBN1*	NM_000138	exon33	c.4081_4082delinsAA	p.Cys1361Asn	NA	Likely Pathogenic	
*FBN1*	NM_000138	exon49	c.6000C>A	p.Cys2000Ter	NA	Pathogenic	
*FBN1*	NM_000138	exon49	c.4544_4546delinsAGAT	p.Pro1515fs	NA	Pathogenic	
*FBN1*	NM_000138	intron21	c.2540-2A>G		NA	Pathogenic	
*FBN1*	NM_000138	intron49	c.6037+2T>C		NA	Pathogenic	
*FBN1*	NM_000138	exon24	c.2740T>C	p.Cys914Arg	NA	Likely Pathogenic	
*FBN1*	NM_000138	exon16	c.1884C>A	p.Cys628Ter	NA	Pathogenic	12068374
*FBN1*	NM_000138	exon15	c.1794C>A	p.Cys598Ter	NA	Pathogenic	
*FBN1*	NM_000138	exon53	c.6446A>G	p.Tyr2149Cys	NA	Likely Pathogenic	24793577
*FBN1*	NM_000138	intron27	c.3337+1G>A		De novo	Pathogenic	
*FBN1*	NM_000138	exon45	c.5434T>C	p.Cys1812Arg	De novo	Likely Pathogenic	19533785
*FBN1*	NM_000138	intron16	c.1960+1delG		De novo	Pathogenic	
*FBN1*	NM_000138	exon45	c.5455C>T	p.Gln1819Ter	NA	Pathogenic	
*FBN1*	NM_000138	exon35	c.4331G>A	p.Cys1444Tyr	NA	Likely Pathogenic	
*FBN1*	NM_000138	exon21	c.2433C>G	p.Cys811Trp	NA	Likely Pathogenic	15241795
*FBN1*	NM_000138	exon64	c.7868dupA	p.His2623fs	Inherited from mother	Pathogenic	
*FBN1*	NM_000138	exon48	c.5873G>A	p.Cys1958Tyr	NA	Likely Pathogenic	21907952
*FBN1*	NM_000138	exon63	c.7711T>C	p.Cys2571Arg	NA	Likely Pathogenic	16222657
*FBN1*	NM_000138	exon56	c.6867T>A	p.Cys2289Ter	NA	Pathogenic	
*FBN1*	NM_000138	intron28	c.3464-2A>G		NA	Pathogenic	
*FBN1*	NM_000138	exon12	c.1374T>A	p.Tyr458Ter	De novo	Pathogenic	
*FBN1*	NM_000138	exon40	c.4897T>C	p.Cys1633Arg	NA	Likely Pathogenic	
*FBN1*	NM_000138	exon11	c.1285C>T	p.Arg429Ter	NA	Pathogenic	11933199
*FBN1*	NM_000138	exon17	c.1968_1969dupCA	p.HisiSer656fs	NA	Pathogenic	
*FBN1*	NM_000138	exon13	c.1561_1562insCAGA	p.Ser521fs	NA	Pathogenic	
*FBN1*	NM_000138	exon35	c.4292G>A	p.Cys1431Tyr	NA	Likely Pathogenic	21542060
*FBN1*	NM_000138	intron48	c.5918-1G>A		De novo	Pathogenic	
*FBN1*	NM_000138	intron48	c.5917+2T>C		NA	Pathogenic	
*FBN1*	NM_000138	exon14	c.1633C>T	p.Arg545Cys	NA	Likely Pathogenic	9338581
*FBN1*	NM_000138	exon9	c.897T>G	p.Cys299Trp	NA	Likely Pathogenic	
*FBN1*	NM_000138	exon7	c.640G>A	p.Gly214Ser	NA	Likely Pathogenic	15733436
*FBN1*	NM_000138	exon45	c.5540G>T	p.Cys1847Phe	Inherited from father	Likely Pathogenic	
*FBN1*	NM_000138	exon64	c.7921C>T	p.Gln2641Ter	NA	Pathogenic	
*FBN1*	NM_000138	intron28	c.3463+1G>T		NA	Pathogenic	
*FBN1*	NM_000138	exon27	c.3217delG	p.Glu1073fs	NA	Pathogenic	
*FBN1*	NM_000138	exon25	c.2987G>A	p.Cys996Tyr	NA	Likely Pathogenic	
*FBN1*	NM_000138	exon56	c.6806T>C	p.Ile2269Thr	NA	Likely Pathogenic	10464652
*FBN1*	NM_000138	exon66	c.8547T>G	p.Tyr2849Ter	NA	Pathogenic	21034599
*FBN1*	NM_000138	exon66	c.6296G>A	p.Cys2099Tyr	NA	Likely Pathogenic	
*FBN1*	NM_000138	exon2	c.3G>A	p.Met1Ile	NA	Pathogenic	
*FBN1*	NM_000138	exon66	c.1098G>C	p.Trp366Cys	NA	Likely Pathogenic	
*FBN1*	NM_000138	exon66	c.5841C>A	p.Cys1947Ter	NA	Pathogenic	
*FBN1*	NM_000138	exon6	c.529T>C	p.Cys177Arg	De novo	Likely Pathogenic	16222657
*FBN1*	NM_000138	exon42	c.5065+1G>A		NA	Pathogenic	17627385
*FBN1*	NM_000138	exon62	c.7636_7642del	p.Gly2546fs	NA	Pathogenic	
*FBN1*	NM_000138	exon3	c.184C>T	p.Arg62Cys	NA	Likely Pathogenic	11826022
*FBN1*	NM_000138	exon34	c.4096G>A	p.Glu1366Lys	NA	Likely Pathogenic	14695540
*FBN1*	NM_000138	exon48	c.5788+1G>A		NA	Pathogenic	11702223
*FBN1*	NM_000138	exon53	c.6431A>G	p.Asn2144Ser	NA	Likely Pathogenic	8504310
*MYH11*	NM_001040114	intron33	c.4599+1G>A		NA	Pathogenic	21937134
*SLC2A10*	NM_030777	exon2	c.1053_1054del	p.Ser351fs	NA	Pathogenic	
*TGFBR1*	NM_004612	exon9	c.1459C>T	p.Arg487Trp	NA	Likely Pathogenic	16928994
*TGFBR1*	NM_004612	exon4	c.678_680del	p.226_227del	De novo	Likely Pathogenic	
*TGFBR2*	NM_001024847	exon7	c.1524dupT	p.Cys508fs	NA	Pathogenic	

NA, not available.

**Table 4:** *FBN1* mutation type and mean average age in patients with various aortic events.

		Aortic dissection	Aortic aneurysm	Valvular disease	Marfan with mild aortic dilation
Truncating	Frameshift insertion	3 (30.0y)	1 (18.0y)	1 (33.0y)	1 (27.0y)
	Frameshift deletion	1 (24.0y)	2 (18.5y)	1 (14.0y)	1 (16.0y)
	Stopgain	6 (33.2y)	3 (24.7y)	2 (31.5y)	1 (17.0y)
Splicing		5 (33.6y)	3 (38.0y)	1 (16.0y)	0
Truncating+Splicing		15 (32.1y)	9 (25.6y)	5 (25.2y)	3 (20.0y)
Missense		13 (36.5y)	12 (33.4y)	1 (17.0y)	1 (39.0y)

y, years old.

**Table 5:** Reclassified variants.

Gene	Transcript	Exon/Intron	Nucleotide change	Protein change	Variant called	Variant reclassification	Reclassification based on	PopFreqMax	Report Ref (PMID)
*COL3A1*	NM_000090	exon48	c.3776C>T	p.Ala1259Val	VUS	Benign	Family segregation	0.0017	22001912
*FBN1*	NM_000138	exon25	c.2953G>A	p.Gly985Arg	Likely Pathogenic	Benign	Family segregation	.	11700157
*FBN1*	NM_000138	exon66	c.8308C>T	p.His2770Tyr	VUS	Benign	Family segregation	0.0001	.
*FBN1*	NM_000138	exon12	c.1427G>A	p.Cys476Tyr	Likely Pathogenic	VUS	Family segregation	.	.
*FBN1*	NM_000138	exon53	c.6380A>G	p.Asp2127Gly	VUS	Benign	Family segregation	.	.
*FBN1*	NM_000138	exon62	c.7627A>C	p.Asn2543His	VUS	Benign	Family segregation	.	.
*FBN1*	NM_000138	exon50	c.6050G>A	p.Cys2017Tyr	Likely Pathogenic	Benign	Family segregation	.	.
*FBN1*	NM_000138	exon59	c.7231G>A	p.Asp2411Asn	VUS	Benign	Family segregation	.	.
*MYH11*	NM_001040114	exon20	c.2293C>A	p.Pro765Thr	VUS	Benign	Family segregation	0.002	.
*MYH11*	NM_001040114	exon31	c.4090G>A	p.Glu1364Lys	VUS	Benign	Family segregation	0.0001	.
*MYLK*	NM_053025	exon10	c.998C>T	p.Pro333Leu	VUS	Benign	Family segregation	.	.
*NOTCH1*	NM_017617	exon34	c.6351C>A	p.Asn2117Lys	VUS	Benign	Family segregation	0.0004	.
*NOTCH1*	NM_017617	exon21	c.3401A>G	p.Gln1134Arg	VUS	Benign	Family segregation	.	.
*NOTCH1*	NM_017617	exon21	c.3402G>C	p.Gln1134His	VUS	Benign	Family segregation	.	.
*SMAD3*	NM_005902	exon1	c.5C>T	p.Ser2Leu	VUS	Benign	Family segregation	.	.
*SMAD3*	NM_005902	exon1	c.147_155del	p.49_51del	VUS	Likely Benign	Family segregation	.	.
*SMAD4*	NM_005359	exon6	c.700A>C	p.Ser234Arg	VUS	Benign	Family segregation	0.00011	.
*TGFBR2*	NM_001024847	exon5	c.1142G>C	p.Arg381Pro	Likely Pathogenic	VUS	Family segregation	.	16283890

VUS, variant of unknown significance.
