# Supplementary material for: Genetic testing of 248 Chinese aortopathy patients using a panel assay
Source: Sci Rep. 2016 Sep 9;6:33002. doi: 10.1038/srep33002 (PMC5017237; doi:10.1038/srep33002)
Supplement: Supplementary Information [file srep33002-s1.doc]

**Genetic testing of 248 Chinese aortopathy patients using a panel assay**

Hang Yang^1^*, Mingyao Luo^2^*, Yuanyuan Fu^1^, Yandong Cao^3^, Kunlun Yin^1^, Wenke Li^1^, Chunjie Meng^1^, Yanyun Ma^1^, Jing Zhang^2^, Yuxin Fan^4^, Chang Shu^2#^, Qian Chang^2#^, Zhou Zhou^1#^

1. State Key Laboratory of Cardiovascular Disease, Beijing Key Laboratory for Molecular Diagnostics of Cardiovascular Diseases, Diagnostic Laboratory Service, Fuwai Hospital, National Center for Cardiovascular Diseases, Chinese Academy of Medical Sciences and Peking Union Medical College, Beijing, 100037, China
2. State Key Laboratory of Cardiovascular Disease, Center of Vascular Surgery, Fuwai Hospital, National Center for Cardiovascular Diseases, Chinese Academy of Medical Sciences and Peking Union Medical College, Beijing, 100037, China
3. Analyses Technologies, Beijing, 100102, China
4. John Welsh Cardiovascular Diagnostic Laboratory, Department of Pediatrics, Baylor College of Medicine, Houston, TX, 77030, USA.

* Contribute equally to the article

#Correspondence to: Chang Shu, E-mail: [changshu@fuwaihospital.org](mailto:changshu@fuwaihospital.org). Tel: (8610)88398188; Qian Chang, E-mail: [chqfw@yahoo.com](mailto:chqfw@yahoo.com). Tel: (8610)88398188; Or Zhou Zhou, E-mail: zhouzhou@fuwaihospital.org. Tel: (8610)88398055.

Supp. Figure S1. Aortopathy panel sequencing performance. A, mean depth; B, coverage.


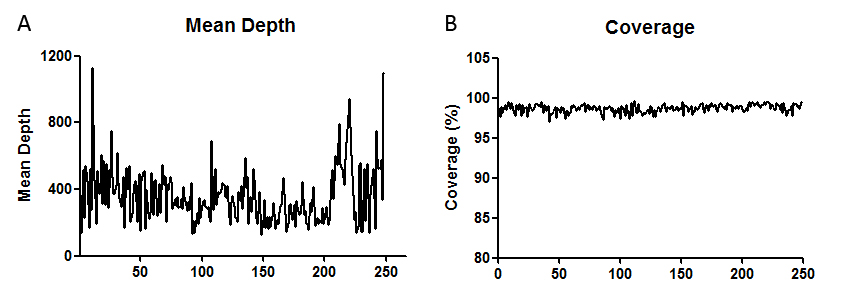


| Supp. Table S1. False negative variants. | | |  |  |
| --- | --- | --- | --- | --- |
| **Gene** | **Transcript** | **Nucleotide change** | **Protein change** | **Pathogenicity** |
| *FBN1* | NM_000138 | c.8308C>T | p.His2770Tyr | VUS |
| *FBN1* | NM_000138 | c.6739+1G>T |  | Pathogenic |
| *FBN1* | NM_000138 | c.3464-2A>G |  | Pathogenic |
| *FBN1* | NM_000138 | c.1969_1970insCA | p.Met657fs | Pathogenic |
| *FBN1* | NM_000138 | c.5918-1G>A |  | Pathogenic |
| *FBN1* | NM_000138 | c.897T>G | p.Cys299Trp | Likely Pathogenic |
| *FBN1* | NM_000138 | c.4847_4848insA | p.Leu1616fs | Pathogenic |
| *FBN1* | NM_000138 | c.5065+1G>A |  | Pathogenic |
| *FBN1* | NM_000138 | c.5788+1G>A |  | Pathogenic |
| *SLC2A10* | NM_030777 | c.1053_1054del | p.Ser351fs | Pathogenic |
| *MYH11* | NM_001040114 | c.4625G>A | p.Arg1542Gln | VUS |
| *MYH11* | NM_001040114 | c.5186C>T | p.Ser1729Leu | VUS |
| *MYH11* | NM_001040114 | c.4208G>A | p.Arg1403Lys | VUS |
| *MYLK* | NM_053025 | c.1976A>G | p.Asn659Ser | VUS |
| *MYLK* | NM_053025 | c.5249A>C | p.Asn1750Thr | VUS |
| *NOTCH1* | NM_017617 | c.2636G>A | p.Arg879Gln | VUS |

VUS, variant of unknown significance.

| Supp. Table S2. Frequent false positive variants removed by iAorta. | | | |
| --- | --- | --- | --- |
| **Gene** | **Transcript** | **Nucleotide change** | **Protein change** |
| *FBN1* | NM_000138 | c.1415G>A | p.Cys472Tyr |
| *FBN2* | NM_001999 | c.2392G>A | p.Gly798Ser |
| *NOTCH1* | NM_017617 | c.3145C>A | p.Gln1049Lys |
| *NOTCH1* | NM_017617 | c.556C>G | p.Pro186Ala |
| *TGFBR2* | NM_001024847 | c.646G>A | p.Val216Ile |
